# Society Meetings

**Published:** 1903-10

**Authors:** 


### SOCIETY MEETINGS.

The American Public Health Association, representing the United States, Canada, Mexico and Cuba, will hold its thirty-first annual meeting at Washington, D. C., Monday, Tuesday, Wednesday, Thursday and Friday, October 26, 27, 28, 29 and 30, 1903, under the presidency of Dr. Walter Wyman, surgeon general of the United States Marine Hospital and Public Health Service, of Washington.

The executive committee has selected the following topics for consideration, but papers will be received upon other sanitary subjects: purification of water supplies; disposal of industrial wastes; purification and disposal of sewage; disposal of garbage; animal diseases and animal food; car sanitation; steamboat and steamship sanitation; etiology of yellow fever; tuberculosis; plague; demography and statistics in their sanitary relation; cause and prevention of infectious diseases; infectious period of communicable diseases; public health legislation; cause and prevention of infant mortality; disinfectants and disinfection; production and free distribution of vaccine; teaching of hygiene and granting of diploma of doctor of public health; sanitary aid societies; canteen in armies.

---

The Buffalo Academy of Medicine held meetings during the month of September, 1903, as follows:

*Section on Medicine.*—Tuesday, September 15. Program: Lake and river current observations, and their value to our city, George E. Fell; The Hamburg filter plant and filtration of water in Holland, A. L. Benedict. Discussion: Drs. Henry R. Hopkins, Walter D. Greene and Wm. G. Bissell.

*Section on Obstetrics and Gynecology.*—Tuesday, September 22. Program: Some practical points in the management of ordinary labor, Peter W. Van Peyma. Discussion: Drs. Ludwig Schroeter and Stephen Y. Howell.

---

The Medical Society of the State of New York, will hold an autumn meeting at the New York Academy of Medicine, 17 West 43d Street, New York City, Tuesday and Wednesday, October 13 and 14, 1903, under the presidency of Dr. A. T. Bristow, Brooklyn. The members of the medical profession are cordially invited to attend.

---

The Medical Association of Central New York, held its 36th annual meeting at Auburn, N. Y., September 22, 1903, under the presidency of Dr. John L. Heffron, of Syracuse. The officers elected for the ensuing year are: president, Dr. Charles A. Van der Beek, Rochester; first vice-president, Dr. Charles G. Stockton, Buffalo; second vice-president, Dr. H. H. Brown, Auburn; secretary, Dr. C. A. Greenleaf, Rochester; treasurer, Dr. William M. Brown, Rochester. The papers read at the meeting will be printed in this Journal.

